# Supplementary material for: Transcriptome Analysis Reveals Vimentin-Induced Disruption of Cell–Cell Associations Augments Breast Cancer Cell Migration
Source: Cells. 2022 Dec 13;11(24):4035. doi: 10.3390/cells11244035 (PMC9776984; doi:10.3390/cells11244035)
Supplement: Supplementary file 1 [file cells-11-04035-s001.zip › DEGs and enrichment analysis.zip!/DEGs and enrichment analysis/WT_VIMvsVector KEGG/GSEA/WT_VIM/CITRATE_CYCLE__TCA_CYCLE_/CITRATE_CYCLE_(TCA_CYCLE)(HSA00020).html]

Details for gene set CITRATE\_CYCLE\_(TCA\_CYCLE)(HSA00020)[GSEA]

|  || Dataset | fpkm.sample |
| Phenotype | sample.cls |
| Upregulated in class | WT\_VIM |
| GeneSet | CITRATE\_CYCLE\_(TCA\_CYCLE)(HSA00020) |
| Enrichment Score (ES) | 0.6577629 |
| Normalized Enrichment Score (NES) | 1.1569608 |
| Nominal p-value | 0.0 |
| FDR q-value | 0.28392905 |
| FWER p-Value | 0.498 |
Table: GSEA Results Summary

  

Fig 1: Enrichment plot: CITRATE\_CYCLE\_(TCA\_CYCLE)(HSA00020)      
 Profile of the Running ES Score & Positions of GeneSet Members on the Rank Ordered List

  

| PROBE | DESCRIPTION (from dataset) | GENE SYMBOL | GENE\_TITLE | RANK IN GENE LIST | RANK METRIC SCORE | RUNNING ES | CORE ENRICHMENT || 1 | ENSG00000235371 | AL122018.1 |  |  | 443 | Infinity | -0.0073 | Yes |
| 2 | ENSG00000227073 | SDHDP2 |  |  | 1625 | Infinity | -0.0272 | Yes |
| 3 | ENSG00000173599 | PC |  |  | 3844 | 3.214 | -0.0099 | Yes |
| 4 | ENSG00000238084 | AL031284.1 |  |  | 3881 | 3.162 | 0.0434 | Yes |
| 5 | ENSG00000273654 | AC020904.2 |  |  | 4160 | 2.890 | 0.0880 | Yes |
| 6 | ENSG00000224183 | SDHDP6 |  |  | 4385 | 2.739 | 0.1310 | Yes |
| 7 | ENSG00000146701 | MDH2 |  |  | 5311 | 2.271 | 0.1540 | Yes |
| 8 | ENSG00000182054 | IDH2 |  |  | 5915 | 2.058 | 0.1789 | Yes |
| 9 | ENSG00000197444 | OGDHL |  |  | 5947 | 2.048 | 0.2134 | Yes |
| 10 | ENSG00000235485 | AL021877.1 |  |  | 6168 | 1.992 | 0.2436 | Yes |
| 11 | ENSG00000067829 | IDH3G |  |  | 6496 | 1.924 | 0.2709 | Yes |
| 12 | ENSG00000101365 | IDH3B |  |  | 6527 | 1.918 | 0.3031 | Yes |
| 13 | ENSG00000073578 | SDHA |  |  | 6709 | 1.875 | 0.3320 | Yes |
| 14 | ENSG00000234213 | FHP1 |  |  | 6788 | 1.858 | 0.3624 | Yes |
| 15 | ENSG00000108958 | AC130689.1 |  |  | 6980 | 1.820 | 0.3902 | Yes |
| 16 | ENSG00000105953 | OGDH |  |  | 7147 | 1.784 | 0.4178 | Yes |
| 17 | ENSG00000131473 | ACLY |  |  | 7230 | 1.767 | 0.4466 | Yes |
| 18 | ENSG00000150768 | DLAT |  |  | 7520 | 1.712 | 0.4709 | Yes |
| 19 | ENSG00000100412 | ACO2 |  |  | 8076 | 1.613 | 0.4890 | Yes |
| 20 | ENSG00000062485 | CS |  |  | 8325 | 1.574 | 0.5116 | Yes |
| 21 | ENSG00000131828 | PDHA1 |  |  | 8691 | 1.521 | 0.5314 | Yes |
| 22 | ENSG00000117118 | SDHB |  |  | 9821 | 1.380 | 0.5358 | Yes |
| 23 | ENSG00000119689 | DLST |  |  | 10265 | 1.328 | 0.5510 | Yes |
| 24 | ENSG00000181227 | DLSTP1 |  |  | 10286 | 1.326 | 0.5733 | Yes |
| 25 | ENSG00000091483 | FH |  |  | 11303 | 1.236 | 0.5772 | Yes |
| 26 | ENSG00000163541 | SUCLG1 |  |  | 12992 | 1.102 | 0.5674 | Yes |
| 27 | ENSG00000100889 | PCK2 |  |  | 13111 | 1.094 | 0.5840 | Yes |
| 28 | ENSG00000014641 | MDH1 |  |  | 13374 | 1.074 | 0.5979 | Yes |
| 29 | ENSG00000168291 | PDHB |  |  | 13581 | 1.058 | 0.6125 | Yes |
| 30 | ENSG00000143252 | SDHC |  |  | 14160 | 1.014 | 0.6200 | Yes |
| 31 | ENSG00000122729 | ACO1 |  |  | 14880 | 0.977 | 0.6245 | Yes |
| 32 | ENSG00000185485 | SDHAP1 |  |  | 16116 | 0.912 | 0.6191 | Yes |
| 33 | ENSG00000136143 | SUCLA2 |  |  | 16391 | 0.894 | 0.6297 | Yes |
| 34 | ENSG00000234115 | AL138878.2 |  |  | 16413 | 0.893 | 0.6446 | Yes |
| 35 | ENSG00000091140 | DLD |  |  | 16530 | 0.886 | 0.6578 | Yes |
| 36 | ENSG00000185986 | SDHAP3 |  |  | 18059 | 0.796 | 0.6455 | No |
| 37 | ENSG00000204370 | SDHD |  |  | 18757 | 0.756 | 0.6466 | No |
| 38 | ENSG00000172340 | SUCLG2 |  |  | 19981 | 0.690 | 0.6376 | No |
| 39 | ENSG00000250767 | AC139783.1 |  |  | 23438 | 0.481 | 0.5873 | No |
| 40 | ENSG00000138413 | IDH1 |  |  | 24628 | 0.388 | 0.5738 | No |
| 41 | ENSG00000236349 | SUCLG2P2 |  |  | 25317 | 0.321 | 0.5676 | No |
| 42 | ENSG00000267162 | SDHDP1 |  |  | 27998 | 0.000 | 0.5222 | No |
| 43 | ENSG00000124253 | PCK1 |  |  | 29111 | 0.000 | 0.5034 | No |
| 44 | ENSG00000272182 | AC135507.1 |  |  | 29796 | 0.000 | 0.4918 | No |
| 45 | ENSG00000264444 | SDHCP1 |  |  | 31436 | NaN | 0.4643 | No |
| 46 | ENSG00000229593 | SUCLA2P3 |  |  | 32649 | NaN | 0.4439 | No |
| 47 | ENSG00000270969 | AP001823.1 |  |  | 32895 | NaN | 0.4399 | No |
| 48 | ENSG00000234946 | SDHCP3 |  |  | 39638 | NaN | 0.3260 | No |
| 49 | ENSG00000217585 | AL021407.2 |  |  | 39672 | NaN | 0.3256 | No |
| 50 | ENSG00000225017 | AP004289.1 |  |  | 39741 | NaN | 0.3246 | No |
| 51 | ENSG00000249226 | SUCLG2P4 |  |  | 39872 | NaN | 0.3226 | No |
| 52 | ENSG00000244381 | AC007849.2 |  |  | 40248 | NaN | 0.3164 | No |
| 53 | ENSG00000215837 | SDHAP2 |  |  | 41277 | NaN | 0.2992 | No |
| 54 | ENSG00000228872 | AC096664.1 |  |  | 42602 | NaN | 0.2769 | No |
| 55 | ENSG00000225477 | AC099730.1 |  |  | 43607 | NaN | 0.2601 | No |
| 56 | ENSG00000270772 | PDHA1P1 |  |  | 44992 | NaN | 0.2368 | No |
| 57 | ENSG00000227427 | Z93019.1 |  |  | 48688 | NaN | 0.1744 | No |
| 58 | ENSG00000214549 | SDHCP2 |  |  | 50701 | NaN | 0.1405 | No |
| 59 | ENSG00000229744 | AC011998.2 |  |  | 51055 | NaN | 0.1347 | No |
| 60 | ENSG00000216352 | AL590824.1 |  |  | 52187 | NaN | 0.1158 | No |
| 61 | ENSG00000163114 | PDHA2 |  |  | 52731 | NaN | 0.1067 | No |
| 62 | ENSG00000224936 | SUCLA2P1 |  |  | 53429 | NaN | 0.0951 | No |
| 63 | ENSG00000226058 | AC092570.1 |  |  | 53603 | NaN | 0.0923 | No |
| 64 | ENSG00000241218 | AC063944.2 |  |  | 54966 | NaN | 0.0695 | No |
| 65 | ENSG00000228367 | AC108120.1 |  |  | 56934 | NaN | 0.0363 | No |
| 66 | ENSG00000277670 | AC098657.3 |  |  | 57136 | NaN | 0.0331 | No |
| 67 | ENSG00000255035 | SDHCP4 |  |  | 57149 | NaN | 0.0331 | No |
| 68 | ENSG00000233107 | SUCLA2P2 |  |  | 57288 | NaN | 0.0309 | No |
| 69 | ENSG00000242222 | AC092989.1 |  |  | 58079 | NaN | 0.0177 | No |
| 70 | ENSG00000236930 | AC099782.1 |  |  | 58372 | NaN | 0.0129 | No |
Table: GSEA details [plain text format]

  

Fig 2: CITRATE\_CYCLE\_(TCA\_CYCLE)(HSA00020)      
 Blue-Pink O' Gram in the Space of the Analyzed GeneSet

  

Fig 3: CITRATE\_CYCLE\_(TCA\_CYCLE)(HSA00020): Random ES distribution      
 Gene set null distribution of ES for **CITRATE\_CYCLE\_(TCA\_CYCLE)(HSA00020)**
